# A prospective study of microbiological characterization and clinical facets of *Stenotrophomonas maltophilia* infections

**DOI:** 10.18502/ijm.v12i4.3934

**Published:** 2020-08

**Authors:** Suvayu Biswas, Anupam Berwal, Kiran Chawla

**Affiliations:** Department of Microbiology, Kasturba Medical College Manipal, Manipal Academy of Higher Education, Manipal, Karnataka, India

**Keywords:** *Stenotrophomonas maltophilia*, Antibiotic resistance, Minimum inhibitory concentration, Biofilm

## Abstract

**Background and Objectives:**

*Stenotrophomonas maltophilia* is a multidrug resistant opportunistic pathogen, which is normally present in hospital settings and has very high mortality rates.

**Materials and Methods:**

A prospective study was conducted over a period of two years. The specimens were processed by Gram staining and aerobic culture. The bacteria were isolated using standard protocols. The extent of antibiotic resistance of commonly used antimicrobials and biofilm production were studied in the isolates.

**Results:**

A total of 80 strains of *Stenotrophomonas maltophilia* were isolated. The maximum sensitivity (93.8%) of these isolates was noticed for cotrimoxazole. 63.7% of strains were strong biofilm producers. The group given pathogen specific antibiotic showed better prognosis (P value ≤ 0.05).

**Conclusion:**

Early diagnosis and proper management of cases infected with *Stenotrophomonas maltophilia* is important to avoid therapeutic failures.

## INTRODUCTION

*Stenotrophomonas maltophilia* is a ubiquitous, aerobic, non-fermentative, biofilm producing Gram negative bacillus which bears a close resemblance to the *Pseudomonas* species and is often linked with the respiratory infections in humans (1). The spectrum of infections linked with *S. maltophilia* include respiratory tract infections, bacteremia, biliary sepsis, infections of the bones and joints, urinary tract, soft tissues, eye infections (keratitis, scleritis, dacryocystitis, endophthalmitis), endocarditis and meningitis (2). Suction tubes, nebulizing equipment, endoscope, haemodialysis samples, faucet, sink drain and shower heads represent a nidus of infection (3). It is an important hospital acquired organism with crude mortality rates ranging from 14% to 69% in patients with bacteremia (2). The predominance of *S. maltophilia* infections is mainly seen in patients with a compromised immune system especially those with hematological malignancies, transplantation of organs, prolonged hospitalization, mechanical ventilation, HIV infection, cystic fibrosis, patients in critical care, those having central venous catheter or other invasive devices (vascular, urinary, biliary), corticosteroids or immunosuppressant therapy and patients previously treated with antibiotics (4).

*S. maltophilia* has the potential to produce biofilms on biotic and abiotic surface, including pneumocytes and is inherently resistant to a wide variety of antibiotics (5). Due to inherent resistance to beta lactam antibiotics, carbapenems, quinolones and aminoglycosides (6), the therapeutic options for *Stenotrophomonas* are very limited. Resistance to carbapenems is by zinc – dependent, chromosomally mediated beta-lactamases; whereas resistance to quinolones and aminoglycosides is accounted to acetyl-transferases and temperature-dependent changes in lipopolysaccharides; and overexpression of energy-dependent efflux pumps may lead to development of resistance in several other drugs (1, 7–11). Currently, the drug of choice for *S. maltophilia* infections is a high-dose cotrimoxazole (4, 8–10), but development of its resistance due to the efflux pumps is a matter of concern for clinicians. Due to its ability to become multidrug resistant (MDR) and extensively drug resistant (XDR), recent WHO classification has tagged *S. maltophilia* as one of the harbingers of organisms which are resistant to multiple drugs (MDRO) in healthcare environment (12). Keeping in view the importance of the *S. maltophilia* as an emerging pathogen in hospital acquired infections and its ability to show multidrug resistance, a prospective study was planned to understand in details the clinico-microbiological profile of *S. maltophilia*.

## MATERIALS AND METHODS

The study is a hospital based prospective observational study which was carried out in the Department of Microbiology at a tertiary care hospital in Karnataka over a period of 24 months (1^st^ October ‘2014–30^th^ September ‘2016). All the patients’ samples whose cultures grew *S. maltophilia* during the study period were included in the study.

### Processing of samples and identification of study isolates.

Culture for all the samples was done on sheep blood agar and MacConkey agar followed by incubation at 37°C for 18–24 hours in 5–10% CO_2_. Laboratory identification of the isolates was carried out using standard biochemical testing and was confirmed with MALDI – TOF MS analysis (Vitek MS Shimadzu bioMerieux).

### Antimicrobial susceptibility testing.

Antimicrobial susceptibility testing was performed according to Clinical Laboratory Standards Institute (CLSI) guidelines (13) by Kirby Bauer disk diffusion method using the following antibiotic discs (Span Diagnostics Ltd, Surat, India) - cotrimoxazole (1.25/23.75 μg), ciprofloxacin (5 μg), cefoperazone-sulbactam (75/30 μg), piperacillin-tazobactam (100/10 μg), netilmicin (30 μg), amikacin (10 μg), gentamicin (10 μg), imipenem (10 μg), aztreonam (10 μg). *Pseudomonas aeruginosa* ATCC 27853 was used as quality control strain. Considering the inherent resistance of *S. maltophilia* to beta lactam antibiotics, quinolones, carbapenems and aminoglycosides, final reporting of sensitivity to clinicians was done for cotrimoxazole and levofloxacin.

### Minimum inhibitory concentration (MIC) test using Epsilometer-strip.

We have chosen three antibiotics for MIC testing because of financial constraints and also because these antibiotics are the preferred antibiotics for treatment. E test strips were procured from bioMerieux ( Marcy l’Etoile, France). The strips were stored in the refrigerator at 2–8°C when not in use. For MIC testing, the E strips were first brought at room temperature. Bacterial suspension was made from 2–4 colonies of the test isolate and were inoculated into peptone water for 4 hours at 35 ± 2°C. The bacterial suspension was adjusted to 0.5 McFarland Standard by diluting with peptone water and inoculated onto Mueller Hinton Agar (MHA) plates by lawn culture. MIC was determined for ceftazidime, ciprofloxacin and cotrimoxazole using E strip method. A single E strip was placed at the centre of the lawn cultured plate of MHA with the help of a sterile forcep. The MHA plates were incubated for 24–48 hours at 37°C. The MIC for the *S. maltophilia* isolates was interpreted following the CLSI guidelines 2012 (13).

### Detection of biofilm production.

Trypticase soy broth (TSB) medium with 1% glucose was prepared and the cultures were inoculated and incubated at 37°C for 24 hours. With a fresh TSB with 1% glucose medium, a 1:100 dilution was made. 0.2 ml of this dilution was added in the wells of 96 well polystyrene flat bottom tissue culture plate and incubated at 37°C for 24 hours. The broth was removed by tapping and the wells were washed with 0.2 ml phosphate buffer saline (pH 7.2) four times. To fix the biofilm, 2% sodium acetate was added to the wells followed by staining with 0.1% w/v crystal violet. Then, excess stain was removed followed by washing with deionized water. After drying the plates, optical density was measured at 570 nm. Isolates were divided into weak, moderate and high biofilm producers based on the optical density values as < 0.120, 0.120–0.240 and > 0.240 respectively.

### Statistical evaluation.

Data analysis was done using SPSS version 16. For demographic data, descriptive analysis was used and results represented as frequency (percentages). Continuous variables were expressed as mean ± standard deviation (SD). Independent Sample t-test was utilised to analyse the duration of hospital stay of overall and ICU patients. Biofilm production and antibiogram data was represented using frequency (percentages). All reported values were two sided (α = 0.05) with a confidence interval of 95%. P value <0.05 was considered significant.

## RESULTS

In the study period, 80 isolates of *S. maltophilia* were identified from various samples, with maximum number from sputum (n = 41, 51.25%) followed by endotracheal aspirate (n = 21, 26.25%), blood (n = 9, 11.25%), wound swab (n = 4, 5%), two each (2.5%) from BAL and pus and one (1.25%) from the tissue. Male to female ratio of study cases was 52:28. Most of the patients (n = 38, 47.5%) were in the age group of 41–60 years. Predominant presenting symptoms were observed as respiratory tract infections (81.3%). The detailed clinico-demographic profile of the study population is shown in Table 1.

**Table 1.** Demographic and clinical details of study population

**Characteristics**	**Cases**
**Gender**	
Male	52 (65%)
Female	28 (35%)
**Age of Overall study population (mean ± SD)**	54 ± 17.454 years
**Presenting symptoms**	
Respiratory tract infections	81.3%
Blood stream infection	16.3%
Skin/soft tissue infection	10%
Central nervous system infection	7.5%
Others	18.9%
**Mortality**	9 (11.25%)
**Co-morbidities**	
Pulmonary disease	64
Hypertension	26
Cardiac disease	25
Diabetes mellitus	25
Renal disease	11
Liver disease	7
Malignancy	5
Hypothyroidism	4
Benign prostatic hyperplasia	3
Neurological disease	2

*S. maltophilia* was isolated as a single pathogen in 48 patients (60% of the cases). The other pathogens that grew with this organism in descending order were *Acinetobacter baumannii* (15%), *Klebsiella pneumoniae* (6.3%), *Pseudomonas aeruginosa* (6.3%), *Enterococcus* spp. (3.8%), *Staphylococcus aureus* (2.5%) followed by *Escherichia coli*, *Moraxella* spp., *Aspergillus* spp., *Enterobacter cloacae*, *Serratia* spp. all contributing to 1.3%.

**Fig. 1.**
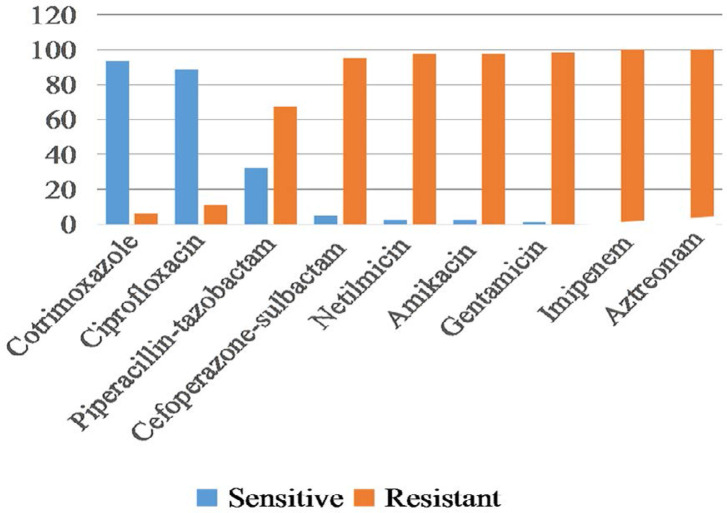
Susceptibility pattern of *S. maltophilia* by Kirby-Bauer disk diffusion method

Results of susceptibility testing done by Kirby-Bauer disk diffusion method revealed that cotrimoxazole (93.8%) was the most sensitive antibiotic followed by ciprofloxacin (88.8%) and piperacillin-tazobactam (32.5%) (Fig. 1).

**Fig. 2.**
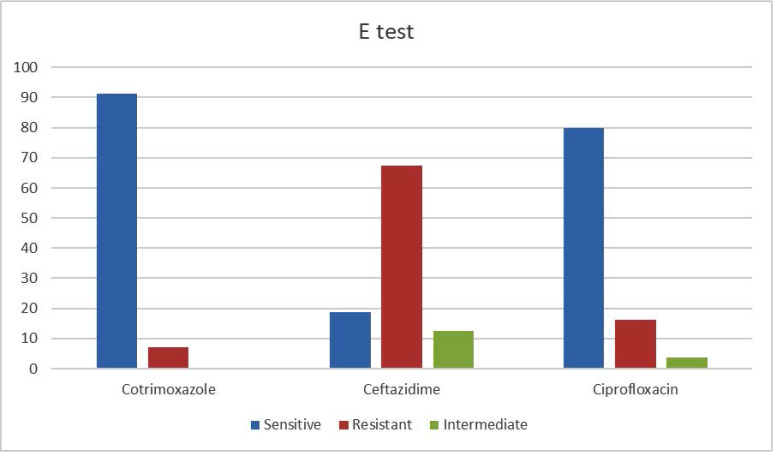
Result of antibiotic sensitivity by E strip method.

The results of E-strip test method are shown in Fig. 2, which has replicated our disk diffusion results. MIC detection was done for only three antibiotics – cotrimoxazole, ceftazidime and ciprofloxacin, because only these antibiotics are used for treatment.

The results of biofilm production revealed 63.7% were strong biofilm producers and 36.3% moderate biofilm producers. Among 80 patients, 39 (48.75%) were on empirical antibiotic therapy and the most common empirical antibiotic used was meropenem followed by β-lactam - β-lactamase combinations, ceftriaxone, amikacin etc. Pathogen specific antibiotic was instituted in 43 (53.75%) patients whereas 37 (46.25%) patients received non pathogen specific antibiotic. Table 2 shows comparative analysis of prognostic indicators between two groups.

**Table 2.** Comparative analysis of group of patients given pathogen specific antibiotics versus those without pathogen specific antibiotics.

	**Frequency N (%)**	**Duration of stay (Mean ± SD)**	**Mortality rate N (%)**
Pathogen specific antibiotic	43 (53.75)	16.03 ± 1.60	1 (3.03)
Non pathogen specific antibiotics	37 (46.25)	19.95 ± 2.40	8 (17.02)
p value^*^	------------	0.054	0.051

* = p value ≤ 0.05 was considered as significant.

## DISCUSSION

In the present study with a sample size of 80, males contributed 65% with the male: female ratio 52:28. The predominance of males is probably due to the behavioural and socioeconomic factors in India, where males in larger proportion to females are involved with outdoor activities and females often don’t present in the early course of the illness.

Present study reports that almost half (N=38, 47.5%) of our patients were between the age group of 41–60 years and about 27 (33.7%) patients were in age group of 61–80 years. This can be attributed to a weekend immune system response observed in older age group which rendered them more susceptible to infections by *S. maltophilia*. Our study is in close association with a study done by Gopalakrishnan R that also showed the mean age group of infected patients as 62.4 years (14).

Present study shows that respiratory tract is the most common system involved (81.3%) followed by blood stream infection (16.3%) and skin/soft tissue infection (10%). The predominance of respiratory tract involvement is also cited by Brooke et al. in their review paper (2). Pulmonary diseases like chronic obstructive pulmonary diseases, bronchiectasis and acute respiratory distress syndrome have been found to be the most frequent comorbidities in our study. Chronic inflammation in these conditions contributes to the lung damage thereby compromising the innate and adaptive immune responses (15) and pre-disposing such cases to infection by *S. maltophilia*.

Bacteremia was observed in 16.3% cases in our study but this is in discordance with a study done by Jang et al. where the blood culture was positive in 68.4% of the cases (16). This discordance may be due to different selection of cases. Studies have reported that previous treatment with anti-pseudomonal antibiotics are risk factors for the development of bacteremia caused by *S. maltophilia* but there are conflicting reports of such evidence (17, 18).

Our study reports maximum susceptibility of *S. maltophilia* to co-trimoxazole (93.8%), followed by ciprofloxacin (88.8%) and piperacillin-tazobactam (32.5%). Another study done by Rutter et al. (19), showed 91% of *S. maltophilia* were susceptible to cotrimoxazole and 62% were sensitive to ciprofloxacin. In our previous study (20), we found 72.7% of strains were sensitive to cotrimoxazole and 78.8% strains sensitive to ciprofloxacin. The study done by Madi et al. (21) showed 100% sensitivity of *S. maltophilia* towards both cotrimoxazole and ciprofloxacin. According to Nayyar et al. (22), sensitivity of cotrimoxazole and ciprofloxacin was 91.3% and 80% respectively towards *S. maltophilia.* A recent study done by Mario Gajdacs et al. revealed that sensitivity of cotrimoxazole and ciprofloxacin was 87.9% and 91.01% respectively (23).

Likewise, the management of the cases infected by *S. maltophilia* vary in different study. Cikman et al. found levofloxacin to be the most effective antibiotic and cited that cotrimoxazole, chloramphenicol, piperacillin-tazobactam and ceftazidime are the other antibiotics which can be used for managing such cases (24). Chung HS et al. have shown a greater susceptibility to cotrimoxazole and minocycline (25), the author has also reported that combination therapy especially cotrimoxazole plus moxifloxacin may aid more than monotherapy in inhibiting or killing *S. maltophilia* (25).

The present study has demonstrated better outcome of cases who were given pathogen specific antibiotics (53.75%). Empirical treatment was opted for 46.25% and meropenem, to which *S. maltophilia* is inherently resistant, was administered in most of these cases. Treatment with nonspecific antibiotics may have pre-disposed the patients to get infected by this pathogen. It is very important to give pathogen specific antibiotics as demonstrated in our study (p ≤ 0.05, Table 2), because it has shown to decrease the patient stay in the hospital and also the mortality rate.

Most of the pathogenic strains (63.7% strong and 36.3% moderate) were observed to be moderate or strong biofilm producers. Earlier Flores T et al. have reported that 53% of their isolated strains were moderate/strong biofilm producers (26). Ability of this pathogen to produce biofilm makes it even more resistant to the prescribed antibiotics. Study done by Sun E et al. (27) revealed that levofloxacin and erythromycin acts synergistically in biofilms, therefore, suggesting the role of combined macrolide therapy as an effective alternative treatment for *S. maltophilia* infection. The present study noticed the mortality rate of 11.25% in the study population. In a study done by Jang TN et al. (16), mortality rate is 69%, and another study done by Victor MA et al. (28) reported mortality of 14%. The variation in the mortality depends on the selection of cases and the pre-morbid conditions. Jang et al. included only bacteremic cases of *S. maltophilia*.

To conclude, the study highlights the need for awareness about *S. maltophilia* as an important nosocomial pathogen having capability of inherent resistance to multiple antimicrobials and stresses the importance of its early diagnosis along with timely management with pathogen specific antibiotic for better prognosis of patients.
